# Occupational burnout, job satisfaction and anxiety among emergency medicine doctors in Turkey

**DOI:** 10.12669/pjms.37.3.3363

**Published:** 2021

**Authors:** Leyla Ozturk Sonmez, Mehmet Gul

**Affiliations:** 1 Leyla Ozturk Sonmez, M.D., Ph.D. Department of Emergency Medicine, Beyhekim State Hospital, Konya, Turkey. Department of Physiology, Selcuklu Medical Faculty, Selcuk University, Konya, Turkey; 2 Prof. Mehmet Gul, Department of Emergency Medicine, Meram Medical Faculty, Necmettin Erbakan University, Konya, Turkey

**Keywords:** Anxiety, Doctor, Emergency medicine, Job satisfaction, Occupational burnout

## Abstract

**Objective:**

The objective of the study was to investigate possible differences in the levels of anxiety, burnout and job satisfaction among emergency medicine doctors based on their age, gender, employment duration, job title and institution.

**Methods:**

General practitioners, residents, specialists and faculty members working in emergency departments (ED) in Turkey were invited to participate in this questionnaire-based study through an e-mail link between September 2018 and January 2019. A total of 141 doctors from different cities of Turkey who completely filled the questionnaire with their own will were recruited for the study. The Maslach Burnout Inventory(MBI) was used to measure occupational burnout levels, the State-Trait Anxiety Inventory(STAI) to measure anxiety levels and the Short Form Minnesota Satisfaction Questionnaire(SFMSQ) to measure job satisfaction levels of doctors working in EDs. In assessing MBI; Emotional Exhaustion score(EE) is considered low for 0-11 points, moderate for 12-17 points and high >17 points; Depersonalization score is considered low for 0-5 points, moderate for 6-9 points and high ≥ 10 points; , Feeling of low personal accomplishment(PA) is considered low for 0-21 points, moderate for 22- 25 points and high ≥ 26 points. In Assessing STAI; 20- 49 points were considered low/ moderate anxiety and 50- 80 points considered high/ very high anxiety. In assessing SFMSQ neutral job satisfaction point was reported 3; so individuals are considered extremely dissatisfied/ not satisfied (low) if job satisfaction point <3 and very/ extremely satisfied (high) if job satisfaction point >3.

**Results:**

The mean age of the study group was 33.3 (±7.3) and mean employment duration was 8.37 (±6.89). In the overall study population, the emotional exhaustion(EE) was high while depersonalization(DP) and reduction of personal accomplishment(PA) were detected at medium level in the evaluation of MBI subscales. In evaluating overall study population for STAI, state and trait anxiety scores both showed the presence of mild to moderate anxiety. The overall study population evaluation results for SFMSQ were, high levels of general and intrinsic job satisfaction and low levels of extrinsic job satisfaction was measured. Younger the age and shorter the duration of employment is found to be associated with a significant increase in EE and DP and a significant decrease in PA.

**Conclusion:**

In this study, scale results showed that doctors working in EDs had high levels of occupational burnout and anxiety, while job satisfaction levels were low. In addition, a significant relationship was found between the decrease in “age and employment duration” and the increase in “depersonalization”.

## INTRODUCTION

Anxiety disorder is described as a condition that emerges as a significant functional disorder and distress condition with a fluctuating course.^1^,^2^ As for the term “burnout” is described as “losing power, not making effort situation” and it is considered a vocational danger.^3^,^4^ Anxiety and depressive conditions are the most common psychiatric conditions among emergency medicine (EM) staff. More than 52% of EM staff have moderate to severe anxiety disorders requiring consultancy and referral for support and treatment.^5^ Anxiety and occupational burnout are factors which negatively affect job satisfaction. Job satisfaction is defined as “the condition of emotional content caused by the professional experiences of an individual at the workplace and the values that individual attributes to the job”.^6^ A strong correlation between job satisfaction with anxiety and occupational burnout is defined lately.^7^ Job-related behaviors, such as resignation intention, discontinuism, and job performance are affected by job satisfaction which lead staff loss and deceleration of work permanency.^8^

The presence of occupational burnout, anxiety and decreased job satisfaction are generally observed in idealistic employees with high goals.^2^,^6^,^9^ Thus, anxiety and exhaustion are quite common in individuals working in a high-pressure environment like emergency departments (EDs), where decisions are vital and should be made quickly. Shazad et al. stated the main factor of burnout as the lack of getting support from the work environment and family members.^10^,^11^ Severe comorbidity of depression, anxiety and stress in health care workers was demonstrated by Ashraf et al.^12^

In the literature, many methods, mostly questionnaires, were used to evaluate the occupational burnout, job satisfaction and anxiety levels of employees.^4^,^13^,^14^ Turkish EPs are facing with heavy work overloads because of overcrowding and also with abuse and injury because of patient and/or their relatives violence. The primary objective of the current study was to measure the possible differences of doctors working in EDs in terms of occupational burnout, anxiety and job satisfaction levels based on gender, job title and institution among Turkish emergency physicians.

## METHODS

Ethics Committee approval was obtained from the Necmettin Erbakan University Ethics Board before beginning the study (NEU: 2918/1526). General practitioners, residents, specialists and faculty members working in emergency departments (ED) in Turkey were invited to participate in this questionnaire-based study through an e-mail link between September 2018 and January 2019. A total of 141 doctors from different cities of Turkey who completely filled the questionnaire with their own will were recruited for the study.

A personal data form including the age, gender, employment duration, job title and institution of the doctors practicing in EDs was used to separate the study population into subgroups based on gender, institution and job title. These groups were then compared according to the results of the questionnaires and scales. In the evaluation of burnout, job satisfaction and anxiety levels, the scales that were used have been proven to be valid and reliable: Maslach Burnout Inventory (MBI), State-Trait Anxiety Inventory (STAI) and Short Form Minnesota Satisfaction Questionnaire (SFMSQ).^4^,^13^,^14^

MBI was assessed as emotional exhaustion score(EE), depersonalization score (DP) and feeling of low personal accomplishment(PA). EE was considered low for 0-11 points, moderate for 12-17 points and high >17 points; DP was considered low for 0-5 points, moderate for 6-9 points and high ≥ 10 points and feeling of low PA was considered low for 0-21 points, moderate for 22- 25 points and high ≥ 26 points. STAI was assessed as 20- 49 points were considered low/ moderate anxiety and 50- 80 points considered high/ very high anxiety. For SFMSQ; neutral job satisfaction point was reported 3; so individuals are considered extremely dissatisfied/ not satisfied (low) if job satisfaction point <3 and very/ extremely satisfied (high) if job satisfaction point >3.^4^,^13^,^14^

### Statistical analysis:

Statistical analysis was performed with SPSS, v.23.0 statistical software (SPSS, Inc., Chicago, IL, USA). The categorical variables were described as frequencies and percentages. Continuous variables were presented as mean and standard deviations. Chi-square (χ^2^) tests were used to evaluate the relationship between categorical variables of study subgroups. Independent T test and Mann Whitney U test for the comparison of two groups and Kruskal Wallis analysis for the comparison of more than two groups were the tests used in continuous variables. The Pearson’s correlation coefficients of continuous variables were also calculated. Cronbach’s alpha coefficient was used for the reliability of MBI, STAI 1-2 and SFMSQ test results and all items achieved a greater than 0.7 rate which indicates acceptable internal consistency. P-values below 0.05 were considered statistically significant.

## RESULTS

Demographic data of the study population, including age, sex, institution, job title and employment duration, are presented in Table-I. In the general evaluation of MBI, a higher-than-average emotional EE and DP and a moderate level of PA were detected. In the general evaluation of STAI, state anxiety and trait anxiety scores showed the presence of low to moderate anxiety, which was below the average. Table-II

**Table-I:** Demographic Parameters (n=141).

Parameters	Values
Age (mean± SD)	33.3± 7.3
Sex n (%) (Men/Women)	82 (58. 2)/59 (41. 8)
**Institution n (%)**	
University Hospital	63 (44. 7)
State Hospital	69 (48. 9)
Private Hospital	9 (6. 4)
**Job Title n (%)**	
Faculty Members	15 (10. 6)
Specialists	52 (36. 9)
Resident Doctors	50 (35. 5)
General Practitioners	24 (17)
**Employment Duration** (**year)** **(mean± SD)**	8.37± 6. 89
Faculty Members	9.5±4.5
Specialists	12.1±5.06
Resident Doctors	4±3.1
General Practitioners	2.22±2.5

**Table-II:** Cronbach’s α reliability coefficient results.

Parameters	Cronbach’ s α reliability coefficient
MBI Emotional Exhaustion	0.92
MBI Depersonalization	0.74
MBI Feeling of low personal accomplishment	0.79
STAI 1 State Anxiety	0.94
STAI 2 Trait Anxiety	0.91
SFMSQ General job satisfaction	0.91
SFMSQ Extrinsic job satisfaction	0.82
SFMSQ Intrinsic job satisfaction	0.89

In the general evaluation of SFMSQ, doctors working in EDs were found to be satisfied as regard to general and intrinsic job satisfaction but dissatisfied as regard to extrinsic job satisfaction. General study population parameters of burnout, anxiety and job satisfaction questionnaires are available in Table-III. In subgroup analysis, there was no significant difference in gender and health institution groups between MBI, STAI 1-2 and SFMSQ (p > 0.05 for all parameters).

**Table-III:** Questionnaire and Inventory Scores of the General Population (n=141).

Parameters	Values (Mean±SD)	Interpretation
MBI Emotional Exhaustion	20. 07± 8. 6	High
MBI Depersonalization	9. 09± 4. 52	Moderate
MBI Feeling of low personal accomplishment	21. 53± 5. 9	Moderate
STAI 1 State Anxiety	40. 05± 7. 5	Low/ medium anxiety
STAI 2 Trait Anxiety	45. 4± 6. 1	Low/ medium anxiety
SFMSQ General job satisfaction	3. 18± 0. 71	High
SFMSQ Extrinsic job satisfaction	2. 89± 0. 81	Low
SFMSQ Intrinsic job satisfaction	3, 37± 0, 73	High

MBI Emotional Exhaustion score; low:0-11, moderate:12-17, high >17; Depersonalization score; low: 0-5, moderate: 6-9, high ≥ 10; , high Feeling of low personal accomplishment; low: 0-21, moderate: 22- 25, high ≥ 26, STAI; Low/ moderate anxiety; 20- 49 and high/ very high anxiety; 50- 80, SFMSQ; neutral job satisfaction point: 3, extremely dissatisfied/ not satisfied (low) if job satisfaction point <3, very/ extremely satisfied (high) if job satisfaction point: >3.

In subgroup analysis based on job title, EE was found to be highest at general practitioners’ group, DP and state anxiety were highest at residents’ group and decreased PA was highest at faculty members group (p = 0.022, p = 0.001, p < 0.001, p = 0.013, respectively)**.** Statistical results based on job title are available in Table-IV.

**Table-IV:** Evaluation of exhaustion, anxiety and job satisfaction questionnaires based on job title groups.

	Group 1 General Practitioners (n= 24)	Group 2 Resident Doctors (n= 50)	Group 3 Specialists (n= 52)	Group 4 Faculty Members (n= 15)	p
MBI Emotional Exhaustion	21.13±8.2	21.12±8	20.46±9.3	13.5±6.2	0.022
MBI Depersonalization	9.13±4.6	10±3.9	9.44±4.6	4.8±3.3	0.001
MBI Feeling of low personal accomplishment	18.96±5.2	20.2±5.9	22.7±5.7	25.7±4	<0.001
SFMSQ General job satisfaction	3.04±0.62	3.12±0.76	3.2±0.73	3.51±0.48	0.24
SFMSQ Intrinsic job satisfaction	3.21±0.72	3.32±0.8	3.41±0.72	3.7±0.43	0.33
SFMSQ Extrinsic job satisfaction	2.77±0.65	2.83±0.87	2.89±0.84	3.24±0.65	0.32
STAI 1 State Anxiety	36.5±7.6	42.1±8.5	40.6±5.5	36.6±7.4	0.013
STAI 2 Trait Anxiety	46.2±4.2	47.1±7.3	44.1±4,.8	42.5±6.5	0.13

MBI Emotional Exhaustion score; low:0-11, moderate:12-17, high >17; Depersonalization score; low: 0-5, moderate: 6-9, high ≥ 10; , high Feeling of low personal accomplishment; low: 0-21, moderate: 22- 25, high ≥ 26, STAI; Low/ moderate anxiety; 20- 49 and high/ very high anxiety; 50- 80, SFMSQ; neutral job satisfaction point: 3, extremely dissatisfied/ not satisfied (low) if job satisfaction point <3, very/ extremely satisfied (high) if job satisfaction point:>3.

According to the correlation analysis, as the age and employment duration of EM doctors decreased, EE and DP increased and PA decreased. In addition, it was found that as the employment duration decreased, trait anxiety increased significantly. A significant decrease was detected in general, intrinsic and extrinsic job satisfaction with increasing EE, DP, state and trait anxiety. In addition, there was a significant positive correlation between PA and job satisfaction (p < 0.05 for all parameters). Details of the correlation analysis are shown in Table-V.

**Table-V:** Details of the correlation analysis.

Parameters	Emotional Exhaustion		Depersonalization		Personal Accomplishment		Trait Anxiety		State Anxiety	

	r [95%Cl]	p	r [95%Cl]	P	r [95%Cl]	p	r [95%Cl]	p	r [95%Cl]	p
Age (Year)	-0.26 [-0.411/-0.09]	0.002	-0.395 [-0.517/-0.263]	<0.001	0.325 [0.189/0.458]	<0.001	-0.170 [-0.301/-0.016]	0.043	-0.64 [-0.254/0.125]	0.45
Employment Duration	-0.28 [-0.428/-0.126]	0.001	-0.363 [-0.488/-0.219]	<0.001	0.319 [0.171/0.445]	<0.001	-0.148 [-0.330/0.260]	0.07	-0.160 [-0.149/0.139]	0.6
General job satisfaction	-0.572 [-0.673/-0.435]	<0.001	-0.385 [-0.529/-0.229]	<0.001	0.375 [0.225/0.495]	<0.001	-0.419 [-0.582/-0.210]	<0.001	-0.268 [-0.469/-0.023]	0.001
Intrinsic job satisfaction	-0.573 [-0.686/-0.427]	<0.001	-0.369 [-0.515/-0.218]	<0.001	0.418 [0.275/0.539]	<0.001	-0.436 [-0.600/-0.230]	<0.001	-0.244 [-0.457/0.008]	0.004
Extrinsic Job satisfaction	-0.472 [-0.584/-0.341]	<0.001	-0.341 [-0.477/-0.180]	<0.001	0.252 [0.096/0.387]	<0.001	-0.326 [-0.481/-0.140]	<0.001	-0.255 [-0.426/-0.046]	0.002

## DISCUSSION

“Doctors are at high risk of burnout in general. Due to their stress factors, they are more sensitive to depressive symptoms and can have difficulty meeting patient demands.^15^ Increasing population density, health-consciousness and responsibilities lead to occupational overload for EM doctors in Turkey. Thus, burnout is becoming more common among EM doctors because of the risky nature of the specialization.

In a recent cross-sectional study conducted in China, high EE scores were detected (especially in young doctors); additionally, a reduced sense of PA was higher in doctors with a longer employment duration.^16^ Compatible with these results, Oliveira et al.^17^ measured the burnout degrees of health care workers and found the highest burnout degrees in the doctors who have higher education levels and are in direct communication with the patients. These results show a higher burnout level in doctors compared to other health staff. In our study, EE scores of EM doctors were high while DP and PA scores were measured at medium level. MBI subscale’s results for our study were in agreement with these studies. Rotenstein et al.^18^ evaluated 182 studies in a systematic review that measures the occupational burnout level of doctors and detected high levels of EE, DP and reduced PA. EE scores of EM doctors in Turkey were found to be high, while DP and reduced sense of PA scores were medium in our study. Between these subscales there was no difference between genders and health institutions, but there was in regard to job title. Besides, EE and DP scores were highest in residents and lowest in faculty members. Additionally, reduced sense of PA score was highest in faculty members and specialists; residents and practitioners followed them in order. High EE and DP scores in residents can be explained by the patient overload and paperwork, pressure from superiors, learning stress and long and irregular working hours. The high reduced sense of PA levels found in faculty members can be explained by the stress of responsibility and the necessity of continuing academic progress along with patient overload. Burnout could cause resignation, distractibility and malpractice so the factors that feed burnout should be determined and prevented for a peaceful work place and patient care.

Anxiety negatively effects job satisfaction which leads work related exhaustion in time. Anxiety levels were reported to be higher in health staff compared to the overall study population due to frequent night shifts, less sleep and high workload in addition to other causes.^19^ It was reported that stress and anxiety could be more significant in EM doctors who work in an unpredictable environment and especially in those who are less experienced.^20^ An increase was shown in stress and anxiety parallel to work load especially in young doctors working in EDs.^5^

In our study, for residents who are on duty for the longest hours and have a young mean age among the EM doctors’ group, state anxiety and trait anxiety scores were higher than other groups; this difference was significant for state anxiety (p = 0.013). State and trait anxiety levels were at a low to medium level in the general study population.

Job satisfaction and burnout were shown to be related to many factors such as job type, physical environment of work place and colleagues.^21^ Health care services are difficult and stressful by nature, and thus depression, dissatisfaction and stress ratios are reported frequently among doctors and continue to increase gradually over time.^22^ In one study, a relationship between burnout and job satisfaction was identified as well as the fact that job satisfaction level affected patient care quality.^23^ These findings support a negative relationship between job satisfaction and general occupational burnout. In our study, the increase of burnout and anxiety levels was found to be correlated with the decrease in job satisfaction. Job dissatisfaction in EDs is related to the increasing patient load.^24^ Approximately 84 million citizens accessed EDs in 2017, according to Republic of Turkey Ministry of Health data and this number is nearly 1.3 times higher than the country population.^25^ Job satisfaction and burnout were shown to be related to exhaustion in our study, but there was no change detected due to factors such as gender, institution, job title, age and employment duration. Also, the SMFSQ results of the total study population showed low extrinsic job satisfaction. We think that the main causes of this condition are patient load, time limit pressures, intense work conditions.

In the overall evaluation of individuals, state anxiety and trait anxiety scores showed the presence of low to medium degree of anxiety; extrinsic job satisfaction score was found to be low but general and intrinsic job satisfaction scores were found to be high. Additionally, EE level was found to be high while DP and reduced PA scores showed a medium level. These scores did not show a change according to gender and institution. Burnout and anxiety scores of residents were higher. We think that these results are due to limited experience, patient overload, time limit pressures and intense work conditions.

Although there are many studies evaluating burnout or job satisfaction as a single parameter about healthcare workers, we were unable to find any study in the literature that evaluates burnout, anxiety and job satisfaction in emergency medicine doctors all together and examines the relationship and correlation of these parameters. This study is the first report in this field.

We believe that application of regulations to improve work environment quality in practice, such as providing adequate educational support, making accurate triage (which may decrease patient load), taking necessary safety precautions, increase in salary and establishing working time regulations to ensure adequate rest breaks, will have a positive effect on burnout, anxiety and personal success. Further studies should be done for a better understanding of the factors underlying anxiety, burnout and job satisfaction and to improve work place quality.”

### Limitations of the study:

The main limitations are the small number of participants, data collection from a single country (Turkey), the short duration of the study, and the fact that the study was conducted only on doctors.

## CONCLUSION

Doctors working in EDs showed high levels of occupational burnout and anxiety levels, while job satisfaction levels were low. In addition, a significant relationship was found between the decrease in age and employment duration and the increase in burnout and depersonalization. This finding may point out that especially young doctors may hesitate to take EM as a specialization. Thus, we recommend that workplace regulations be made in physical, administrative and social areas to improve ED working conditions.
